# Reply to ‘Comment on ‘Dairy, calcium, vitamin D and ovarian cancer risk in African–American women’’

**DOI:** 10.1038/s41416-018-0163-1

**Published:** 2018-07-02

**Authors:** Bo Qin, Lauren C. Peres, Joellen M. Schildkraut, Elisa V. Bandera

**Affiliations:** 1 Department of Population Science, Rutgers Cancer Institute of New Jersey, New Brunswick, NJ 08903 USA; 2 Department of Public Health Sciences, University of Virginia, Charlottesville, VA 22908 USA

**Keywords:** Risk factors, Cancer epidemiology

We appreciate the opportunity to respond to Dr. Hilliard’s letter^1^ expressing concerns that our manuscript^2^ is recommending ‘to increase calcium intake among African–American women as a means of reducing their susceptibility to the disease’ (ovarian cancer). This is a misrepresentation of the study’s purpose and conclusions. Our study evaluated the associations of calcium, vitamin D and dairy food intakes with ovarian cancer risk (not cancer susceptibility) among African–American women. We found inverse associations between calcium intake from food, supplement or total (from food plus supplement sources) and the risk of ovarian cancer. We also observed an increased ovarian cancer risk for higher whole-milk consumption and lactose intake. Dr. Hilliard questions our finding on calcium based on the literature on TRPV6 calcium ion channel, and suggests that our findings could not apply to African–American women; because, we used a calcium standard based on European ancestry and ‘selected out a mixed-race population’. However, the literature cited by Dr. Hilliard does not show the relation between TRPV6 and ovarian cancer risk. We also found Dr. Hilliard misrepresented our data and analysis, which we wish to clarify.

To our knowledge, no study has evaluated TRPV6 variants and ovarian cancer risk. In the literature review cited by Dr. Hilliard,^3^ two studies were related to ovarian tissues. The first one, examining two tumour samples, found elevated levels of Ca^2+^ transport protein (Ca^2+^-selective channels in the TRPV channel family) in ovarian adenocarcinoma tissues compared to normal ovarian tissue.^4^ The other one reported that no human Ca^2+^ transport protein-like transcripts were detected in normal ovarian tissue;^5^ this study did not examine ovarian tumour tissues. Furthermore, the other two studies cited by Dr. Hilliard in support of dietary calcium increasing ovarian cancer risk in Black women evaluated impact on metastatic prostate cancer,^6,7^ not the onset of ovarian cancer. Several dietary factors have been shown to have organ-specific effects.^8^ For example, calcium has been shown to be detrimental for prostate cancer but beneficial for colorectal cancer.^9,10^ Therefore, findings from one cancer site cannot be directly extrapolated to another site. However, we understand Dr. Hilliard’s concern that TRPV6 variant may be a confounder or effect-measure modifier that influences the relation between calcium intake and ovarian cancer risk among African–American women. Our preliminary Genome-Wide Association Studies (GWAS) results among African–American women did not reveal any significant association of ovarian cancer with the major non-synonymous polymorphisms of *TRPV6* gene (rs4987657, rs4987667, and rs4987682)^11^ and therefore do not support this hypothesis.

Dr. Hilliard claims that our study design is flawed because we used ‘“one size fits all” methodology’, i.e., using the calcium and vitamin D standards based on European ancestry women, which is not true. The methods we used to define the reference group and the higher intake groups were based on the intake distributions specific to our study sample of African–American ovarian cancer cases and controls. As we described in Methods section, calcium and vitamin D intakes ‘were categorized into quartiles based on the distribution of controls’, which is standard methodology in nutritional epidemiology. If we have understood Dr. Hilliard correctly, the claim regarding ‘the dietary calcium standards that work best for females of Northern European ancestry in protecting them from osteoporosis’ referred to the current recommended daily allowance of calcium intake from Institute of Medicine (IOM), which is 1000 or 1200 mg/day for adult women ≤50 years or women >50 years, respectively.^12^ Although, we did not use these cutoff points for study analyses, our results do not seem to support that calcium intakes at these levels increase ovarian cancer risk; rather our results showed that compared to African–American women who consumed ≤478.6 mg/day of calcium, those in the higher intake groups (Q3: 784.2–1233.6 mg/day and Q4: ≥1233.7 mg/day) had significantly lower odds of ovarian cancer.

Dr. Hilliard further points out design flaws in our statistical analyses for not showing what it purports to show and that our conclusions were not supported by the data provided, which reflects a lack of understanding of multivariable analyses of case-control data on nutrition and cancer epidemiology. In fact, multivariable-adjusted odds ratios are ignored, distributions are discussed instead, and tables are misread. For example, the statement ‘72.7% of these cancer patients…do not drink milk’ is a misrepresentation of our results; as this percentage refers to non-consumers of whole milk only.

Furthermore, we disagree with Dr. Hilliard’s points that our study was ‘selected out of a mixed-race population’ and was subjected to ‘stratification bias’ by race. We conducted a sensitivity analysis among African–American women who had genetic data in our study (229 cases, 455 controls). The proportion of intercontinental ancestry was estimated and a stringent cutoff of >50% African ancestry proportion was used to define African, as described previously.^13,14^ Only ten women had <50% African ancestry, and excluding them from analysis did not alter our findings. Further adjusting the proportion of African ancestry in our models did not materially change the results (Table 1).

**Table 1:** Results of sensitivity analysis adjusting for African ancestry proportion in AACES

	Published results						New sensitivity analysis^a^					
	Cases (*n* = 490)		Controls (*n* = 656)		Multivariate adjusted^b^		Cases (*n* = 226)		Controls (*n* = 448)		Multivariate Adjusted + African ancestry proportion	
	*n*	%	*n*	%	OR	95% CI	*n*	%	*n*	%	OR	95% CI
*Total dairy (serving/wk)*												
Q1 (≤2.6)	103	21.0	168	25.6	1.00	Ref	42	18.6	118	26.3	1.00	Ref
Q2 (2.7–4.7)	144	29.4	167	25.5	1.48	1.04, 2.11	65	28.8	111	24.8	1.81	1.10, 2.98
Q3 (4.8–8.3)	118	24.1	157	23.9	1.32	0.91, 1.92	57	25.2	113	25.2	1.52	0.90, 2.55
Q4 (≥8.4)	125	25.5	164	25.0	1.48	0.95, 2.28	62	27.4	106	23.7	1.72	0.95, 3.11
*p* for trend					0.25						0.27	
*Milk (serving/wk)*												
Q1 (≤0.8)	103	21.0	161	24.5	1.00	Ref	40	17.7	115	25.7	1.00	Ref
Q2 (0.9–1.7)	121	24.7	161	24.5	1.20	0.83, 1.74	55	24.3	107	23.9	1.50	0.89, 2.54
Q3 (1.8–4.1)	139	28.4	173	26.4	1.20	0.83, 1.75	65	28.8	119	26.6	1.46	0.86, 2.49
Q4 (≥4.2)	127	25.9	161	24.5	1.34	0.87, 2.05	66	29.2	107	23.9	1.70	0.94, 3.05
*p* for trend					0.30						0.23	
*Whole milk (serving/wk)*												
Non-consumer	356	72.7	511	77.9	1.00	Ref	173	76.6	347	77.5	1.00	Ref
≤2.3	56	11.4	73	11.1	1.20	0.68, 2.12	20	8.9	53	11.8	0.97	0.54, 1.71
>2.3	78	15.9	72	11.0	1.85	1.05, 3.27	33	14.6	48	10.7	1.50	0.86, 2.63
*p* for trend					0.02						0.16	
*Total calcium (mg/d)*												
Q1 (≤478.6)	134	27.3	164	25.0	1.00	Ref	52	23.0	117	26.1	1.00	Ref
Q2 (478.7–784.1)	142	29.0	164	25.0	0.89	0.61, 1.31	62	27.4	114	25.5	0.85	0.49, 1.45
Q3 (784.2–1233.6)	108	22.0	164	25.0	0.62	0.39, 0.96	57	25.2	109	24.3	0.67	0.36, 1.25
Q4 (≥1233.7)	106	21.6	164	25.0	0.51	0.30, 0.86	55	24.3	108	24.1	0.49	0.24, 1.01
*p* for trend					0.009						0.05	
*Total vitamin D (IU/d)*												
Q1 (≤130.8)	149	30.4	164	25.0	1.00	Ref	52	23.0	115	25.7	1.00	Ref
Q2 (130.9–292.8)	98	20.0	164	25.0	0.72	0.49, 1.04	48	21.2	116	25.9	0.98	0.58, 1.67
Q3 (292.9–523.9)	118	24.1	164	25.0	0.89	0.60, 1.32	64	28.3	115	25.7	1.36	0.78, 2.36
Q4 (≥524.0)	125	25.5	164	25.0	1.00	0.65, 1.54	62	27.4	102	22.8	1.42	0.78, 2.59
*p* for trend					0.60						0.17	
*Lactose (g/d)*												
Q1 (≤2.3)	104	21.22	170	25.9	1.00	Ref	43	19.0	121	27.0	1.00	Ref
Q2 (2.4–4.6)	137	27.96	164	25.0	1.44	1.00, 2.08	55	24.3	112	25.0	1.48	0.87, 2.51
Q3 (4.7–8.8)	108	22.04	158	24.1	1.19	0.79, 1.78	59	26.1	103	23.0	1.56	0.89, 2.72
Q4 (≥8.9)	141	28.78	164	25.0	1.97	1.25, 3.10	69	30.5	112	25.0	1.89	1.02, 3.52
*p* for trend					0.008						0.11	

^a^The sensitivity analysis was conducted among a total of 674 participants who had available genetic data with >50% African ancestry.

^b^Model adjusted for age, region, total energy intake, education, parity, oral contraceptive use, menopausal status, tubal ligation, family history of breast/ovarian cancer, daylight hours spent outdoors in summer months, pigmentation, recreational physical activity, body mass index, plus supplemental calcium intake and other types of dairy products when applicable for dairy products analyses; or plus other sugar intake excluding lactose, quartiles of total calcium, total vitamin D, and lactose when applicable for nutrient intakes analyses

We agree with Dr. Hilliard’s point that ‘no new recommendations should be made for changing the calcium intake of African–American women with or without ovarian cancer’, but for different reasons. First, we did not make recommendations; we reported findings that suggested an inverse association. Second, we did not examine the impact of current recommended levels of calcium intake in our study, but as we mentioned earlier, we found higher calcium intakes (at levels close to the current recommendations) were associated with decreased ovarian cancer risk. To our knowledge, our study represents the first attempt to evaluate the impact of intakes of dairy foods, lactose, calcium, and vitamin D exposure on ovarian cancer risk among African–American women and will need to be replicated in other studies, particularly in cohort studies, before any public health recommendations can be made.
